# A case of Empty Sella syndrome with adrenal insufficiency masked by prednisolone after administration of immune checkpoint inhibitors

**DOI:** 10.1097/MD.0000000000037204

**Published:** 2024-03-08

**Authors:** Yuichiro Iwamoto, Fuminori Tatsumi, Mana Ohnishi, Yukino Katakura, Tomohiko Kimura, Masashi Shimoda, Shuhei Nakanishi, Tomoatsu Mune, Hideaki Kaneto

**Affiliations:** Division of Diabetes, Endocrinology and Metabolism, Kawasaki Medical School, Kurashiki-city, Okayama, Japan.

**Keywords:** adrenal insufficiency, Empty Sella syndrome, hypopituitarism, Immune checkpoint inhibitor, nonsmall cell lung cancer

## Abstract

**Introduction:**

The use of immune checkpoint inhibitors (ICIs) is gradually increasing; ICIs produce a variety of immune-related adverse events (irAEs), especially ICI-induced hypoadrenocorticism, which can be a lethal complication if treatment is delayed.

**Patient concerns:**

A 63-year-old man received chemotherapy with pembrolizumab for nonsmall cell lung cancer. He developed drug-induced interstitial pneumonia 366 days after receiving pembrolizumab and was treated with prednisolone. Five hundred thirty-seven days later, he developed drug-induced eosinophilic enteritis, and pembrolizumab was discontinued and prednisolone was continued. After discontinuation of prednisolone, general malaise and edema of the lower extremities appeared, and adrenal insufficiency was suspected.

**Diagnosis:**

In blood tests on admission adrenocorticotropic hormone (ACTH) was 2.2 pg/mL and cortisol was 15 μg/dL, with no apparent cortisol deficiency. However, the cortisol circadian rhythm disappeared and remained low throughout the day; a corticotropin-releasing hormone stimulation test showed decreased reactive secretion of ACTH. Pituitary magnetic resonance imaging showed pituitary emptying, suggesting Empty Sella syndrome.

**Interventions and outcomes:**

We started hydrocortisone and his symptoms were improved.

**Conclusions:**

The administration of high-dose steroids after ICI administration may mask the symptoms of hypoadrenocorticism as irAEs. Therefore, we should bear in mind the possibility of hypoadrenocorticism when we stop steroid therapy in patients who are treated with steroids after ICI administration.

## 1. Introduction

Immune checkpoint inhibitors (ICIs) that are currently in clinical use include cytotoxic T-lymphocyte-associated protein 4 (CTLA-4) inhibitors, programmed cell death-1 (PD-1) inhibitors, and programmed cell death-1 ligand 1 (PD-L1) inhibitors. Their use is gradually increasing due to the growing number of cancer indications. ICIs produce a variety of immune-related adverse events (irAEs), and the frequency of irAEs related to endocrine tissues, such as hypothyroidism and hypoadrenocorticism, is 5–10%.^[1]^ ICI-induced hypoadrenocorticism is a lethal complication if treatment is delayed, and the disability is generally permanent.^[2]^ On the other hand, steroid therapy is often effective for gastrointestinal irAEs and interstitial pneumonia, which can cause adrenal insufficiency.^[3]^ This case is a case of drug-induced interstitial pneumonia and drug-induced eosinophilic enteritis after administration of pembrolizumab, an ICI, and adrenal insufficiency along with discontinuation of prednisolone for irAEs.

## 2. Case presentation

A 63-year-old man received immunotherapy with pembrolizumab 200 mg/day for treatment of nonsmall cell lung cancer. Prior to pembrolizumab, in blood tests adrenocorticotropic hormone (ACTH) was 35.4 pg/mL, and cortisol was 12.4 μg/dL. He developed destructive thyroiditis 78 days after the first dose of ICI and received levothyroxine 0.75–0.875 μg/day in an endocrine outpatient clinic. The patient developed drug-induced interstitial pneumonia caused by pembrolizumab 366 days after receiving ICI and started taking prednisolone (30 mg/day). Pembrolizumab was continued whereas the steroid dose was reduced, for a total of 4200 mg; 537 days after the first dose of ICI, the patient developed drug-induced eosinophilic enteritis, and pembrolizumab was discontinued. Prednisolone was discontinued 730 days after the first dose of ICI, but he developed general malaise, edema of the lower legs, and no improvement. Thus, he visited the endocrine outpatient clinic 728 days after ICI administration. He was suspected to have adrenal insufficiency due to long-term steroid administration and was admitted to our hospital.

**Figure 1.**
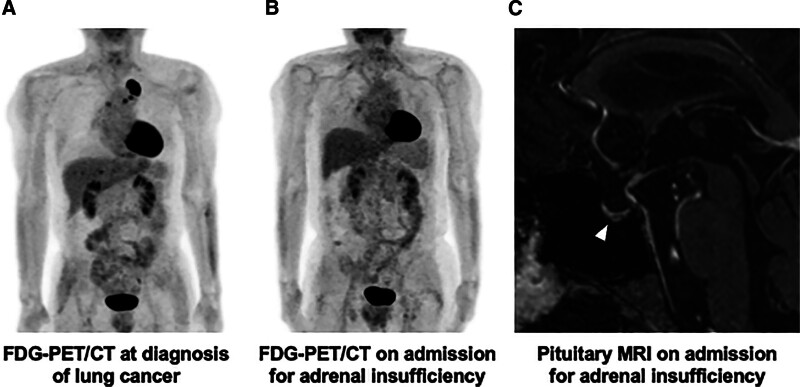
FDG-PET/CT and pituitary MRI in this subject. FDG-PET/CT at the time of diagnosis of lung cancer showed primary lung cancer in the left upper lobe, mediastinal lymph node metastasis and right adrenal metastasis (A), FDG-PET/CT during admission for suspected adrenal insufficiency showed no accumulation in the primary lung cancer, mediastinal lymph nodes, or right adrenal gland (B), and T1-weighted images of gadolinium contrast-enhanced pituitary MRI showed pituitary emptying (C). FDG-PET/CT = 18F-fluorodeoxyglucose positron emission tomography computed tomography, MRI = magnetic resonance imaging.

On admission, his height, body weight, and body mass index were 160.2 cm, 69.2 kg, and 27.0 kg/m^2^. Blood pressure was 133/88 mm Hg, heart rate was 65 beats/min, and body temperature was 36.4°C. There was indurated edema in the lower legs. There was no pigmentation and no other obvious physical findings. The results of blood and urine tests on admission are shown in Table 1. Peripheral blood eosinophil count was 308/μL, with no apparent increase. ACTH was 2.2 pg/mL, and cortisol was 15 μg/dL, with no apparent cortisol deficiency. Thyroid-stimulating hormone was 0.32 μIU/mL, free triiodothyronine (FT3) 3.58 pg/mL, and free thyroxine (FT4) 0.92 ng/dL under oral levothyroxine. Growth hormone (GH) was low at 0.07 ng/mL and somatomedin C 80 ng/mL. The results of diurnal variation evaluated on day 793 after ICI administration and CRH/thyrotropic releasing hormone/luteinizing hormone releasing hormone stimulating tests performed on day 794 after ICI administration are shown in Table 2. The diurnal variation of cortisol disappeared, and cortisol level remained low throughout the day. Corticotropin-releasing hormone (CRH)/thyrotropic releasing hormone/luteinizing hormone releasing hormone stimulation test showed decreased reactive secretion of ACTH and prolactin. 18F-fluorodeoxyglucose positron emission tomography computed tomography (FDG-PET/CT) showed no accumulation in the primary lung cancer lesion and metastatic lesion in the right adrenal gland (Fig. 1A), which was observed at the time of onset (Fig. 1B). Pituitary magnetic resonance imaging (MRI) showed cavitation of the pituitary gland, suggesting Empty Sella syndrome (Fig. 1C). After discontinuation of long-term prednisolone treatment, general malaise and hypoglycemia appeared, and the disappearance of diurnal fluctuation of cortisol suggested adrenal insufficiency. In addition, the patient had a decreased ACTH response in the CRH stimulating test, and the loss of diurnal variation in ACTH and pituitary MRI showed pituitary emptying, suggesting the presence of hypopituitarism. Therefore, we think that this patient had isolated ACTH insufficiency induced by usage of ICI pembrolizumab and adrenal insufficiency due to long-term usage of prednisolone. We started 15 mg/day of hydrocortisone 3 days after admission. Although blood tests showed no obvious symptoms suggestive of adrenal insufficiency, the patient’s general malaise on admission was improved promptly after administration of hydrocortisone, suggesting that decreased secretion of ACTH and cortisol brought about the symptoms.

**Table 1:** Clinical data on admission in this subject.

Peripheral blood		Blood biochemistry		Endocrine examination	
Red blood cells	494 × 10^4^/μL	Total protein	6.1 g/dL	TSH	0.32 μIU/mL
Hemoglobin	14.5 g/dL	Albumin	3.8 g/dL	FT3	3.58 ng/mL
Hematocrit	44.3%	Total bilirubin	0.7 mg/dL	FT4	0.92 ng/mL
White blood cells	6550/μL	AST	20 U/L	ACTH	46.9 pg/mL
Neutrophils	64.8%	ALT	14 U/L	Cortisol	4.4 μg/dL
Lymphocytes	21.4%	γ-GTP	21 U/L	DHEA-S	167 μg/dL
Monocytes	7.9%	LDH	240 U/L	GH	0.07 ng/mL
Eosinophils	4.7%	ALP	49 U/L	IGF-1	80 ng/mL
Basophils	1.2%	ChE	364 U/L	PRL	24.9 ng/mL
Platelet	19.1 × 10^4^/μL	Creatinine	1.08 mg/dL	Thyroid-related autoantibodies	
Electrolytes		BUN	12 mg/dL	TPOAb	<9.0 IU/mL
Sodium	145 mmol/L	UA	7.9 mg/dL	TgAb	83.6 IU/mL
Potassium	4.1 mmol/L	Blood glucose	89 mg/dL	TSAb	109%
Chloride	109 mmol/L	CRP	0.13 mg/dL	TPOAb	<0.8 IU/mL

ACTH = adrenocorticotropic hormone, ADH = antidiuretic hormone, ALP = alkaline phosphatase, ALT = alanine aminotransferase, AST = aspartate aminotransferase, BUN = blood urea nitrogen, CRP = C-reactive protein, FSH = Follicle-stimulating hormone, FT3 = free triiodothyronine, FT4 = free thyroxine, GH = growth hormone, IGF-1 = Insulin-like growth factor, LDH = lactate dehydrogenase, LH = Luteinizing hormone, PRL = prolactin, TgAb = thyroid peroxidase antibody, TPOAb = thyroglobulin antibody, TRAb = thyrotrophin receptor antibody, TSAb = thyroid-stimulating antibody, TSH = thyroid-stimulating hormone, UA = uric acid, γ-GTP = γ-glutamyl transpeptidase.

**Table 2:** Result of circadian rhythm and CRH/TRH/LHRH stimulation test on admission in this subject.

Circadian rhythm	8 o’clock	14 o’clock	20 o’clock	23 o’clock
ACTH (pg/mL)	46.9	18.3	18.5	16.6
Cortisol (μg/dL)	4.4	1.5	1.7	1.6
CRH/TRH/LHRH stimulation test	0 min	30 min	60 min	120 min
ACTH (pg/mL)	46.9	69.5	58.4	32.5
Cortisol (μg/dL)	4.4	8.5	9.4	5.1
TSH (μIU/mL)	0.32	2.20	2.27	1.46
LH (mIU/mL)	5.49	13.13	15.21	15.77
FSH (mIU/mL)	11.15	12.94	14.97	17.10
PRL (ng/mL)	24.9	29.4	29.7	32.5

ACTH = adrenocorticotropic hormone, CRH = corticotropin-releasing hormone, FSH = Follicle-stimulating hormone, LH = Luteinizing hormone, LHRH = luteinizing hormone releasing hormone, PRL = prolactin, TRH = thyrotropic releasing hormone, TSH = thyroid-stimulating hormone.

**Figure 2.**
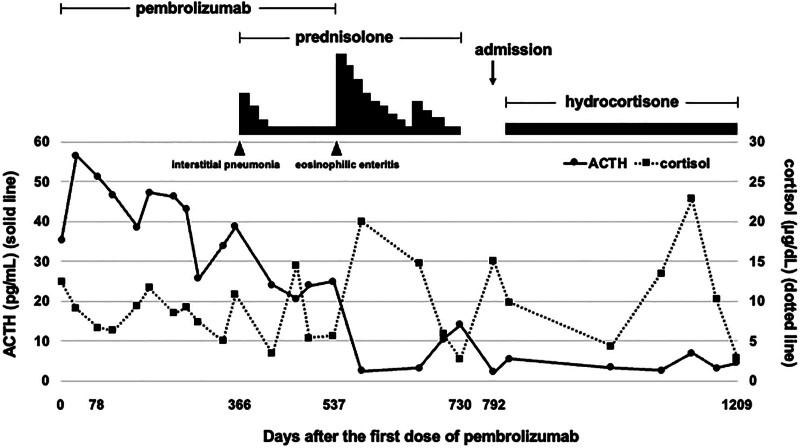
Changes in ACTH and cortisol levels, and steroid dosage after the first dose of pembrolizumab. The solid line indicates ACTH level and corresponds to the values on the left axis. The dotted line shows cortisol level and corresponds to the values on the right axis. The lower axis shows the number of days after the administration of pembrolizumab; day 792 is the date of admission to the endocrinology clinic due to suspected adrenal insufficiency. ACTH = adrenocorticotropic hormone.

Figure 2 shows ACTH and cortisol levels and the course of treatment with steroids from the diagnosis of lung cancer. After discharge from our hospital, the patient has been treated with hydrocortisone and levothyroxine replacement therapy with no recurrence of subjective symptoms. After discharge from our hospital, ACTH has been gradually declining.

## 3. Discussion

The most common cause of immune checkpoint inhibitor-induced hypoadrenocorticism is isolated ACTH insufficiency (92.7%), followed by primary hypoadrenocorticism (5.7%) and mixed type (1.9%).^[4]^ In this case, adrenal insufficiency was suspected because right adrenal metastasis was noted at the time of diagnosis of lung cancer. The patient had been receiving prednisolone for a long time, the diurnal variation of cortisol disappeared, and ACTH was relatively high. On the other hand, in CRH load test, there was insufficient reactive secretion of ACTH, and there was no circadian rhythm of ACTH. It is possible that the pituitary ACTH secretion was temporarily impaired by the effects of long-term administration of high-dose steroids, but outpatient blood tests showed low ACTH levels. We think that hypoadrenocorticism in this patient was induced by an overlap of impaired ACTH secretion due to usage of ICI pembrolizumab and adrenal insufficiency due to long-term usage of prednisolone. In addition, the patient had a decreased response to prolactin stimulation, low absolute thyroid-stimulating hormone levels after stimulation, and low levels of GH and IGF-1 in the early morning, which may have been accompanied by anterior pituitary hypopituitarism. The GH releasing peptide-2 stimulating test could not be performed in this patient due to the presence of a malignant tumor.

Pituitary MRI in this patient showed Empty Sella syndrome. It has been reported that pituitary MRI findings are normal in 93% of isolated ACTH insufficiency by ICI.^[5]^ The incidence of Empty Sella syndrome in the general population has been previously reported to be 12%, with a female-to-male ratio of 5:1.^[6]^ A retrospective study of hypopituitarism by ICI reported enlarged pituitary stalk as a characteristic finding on pituitary MRI.^[1]^ On the other hand, there are a few reports of Empty Sella syndrome induced by ICI,^[7,8]^ and the frequency of this complication is unknown. These 2 case reports of Empty Sella after treatment with ICI were both treated with PD-1 inhibitor. Further studies are needed to determine the impact of Empty Sella syndrome on hypopituitarism induced by ICI and the mechanism of hypopituitarism.

Taken together, we experienced a case of Empty Sella syndrome with adrenal insufficiency masked by prednisolone after administration of ICI. It is likely that this patient had isolated ACTH insufficiency due to irAEs by ICI pembrolizumab and adrenal insufficiency due to long-term usage of prednisolone. The administration of high-dose steroids after ICI administration may mask the symptoms of hypoadrenocorticism as irAEs. It should be kept in mind that adrenal insufficiency may overlap, especially in patients who require prolonged steroid administration due to irAEs by ICI.

## Author contributions

**Writing – original draft:** Yuichiro Iwamoto.

**Writing – review & editing:** Fuminori Tatsumi, Mana Ohnishi, Yukino Katakura, Tomohiko Kimura, Masashi Shimoda, Shuhei Nakanishi, Tomoatsu Mune, Hideaki Kaneto.
